# Molecular Mechanism of Crataegi Folium and Alisma Rhizoma in the Treatment of Dyslipidemia Based on Network Pharmacology and Molecular Docking

**DOI:** 10.1155/2022/4891370

**Published:** 2022-06-08

**Authors:** Hui Wang, Hua Wang, Jin Zhang, Jiahui Luo, Caidong Peng, Xiaoyun Tong, Xudong Chen

**Affiliations:** ^1^The First Affiliated Hospital of Yunnan University of Chinese Medicine, Kunming 650021, Yunnan, China; ^2^Department of Psychiatry, National Clinical Research Center for Mental Disorders, The Second Xiangya Hospital of Central South University, Changsha 410011, Hunan, China

## Abstract

**Background:**

Dyslipidemia has become a critical global issue for public health, with elevating prevalence and morbidity closely related to many cardiovascular diseases (CVD) with high incidence rates. Crataegi Folium (known as Shanzhaye in China, SZ, the leaves of *Crataegus pinnatifida* Bge. var. major N.E. Br. or *Crataegus pinnatifida* Bge) and Alisma rhizoma (known as Zexie in China, ZX, the dried tuber of *Alisma orientale* (Sam.) Juzep or *Alisma plantago-aquatica* Linn), a classic combination of herbs, have been widely used to treat dyslipidemia. However, the therapeutic mechanism of this pair still remains unclear. Hence, this study aimed to elucidate the molecular mechanism of the Shanzhaye-Zexie herb pair (SZHP) in the treatment of dyslipidemia with the use of a network pharmacology analysis approach.

**Methods:**

Active compounds, targets of the SZHP, and targets for dyslipidemia were screened based on the public database. Gene Ontology (GO) and Kyoto Encyclopedia of Genes and Genomes (KEGG) pathway enrichment were performed on the database for annotation, visualization, and integrated discovery (DAVID 6.8). The compound-target-disease-pathway network was visualized using the Cytoscape software, and SYBYL was used for molecular docking.

**Results:**

Twelve active compounds in the SZHP were screened out, which were closely connected to 186 dyslipidemia-related targets. The network analysis revealed that sitosterol, stigmasterol, isorhamnetin, kaempferol, and quercetin might be candidate agents and CCND1, CASP3, HIF1A, and ESR1 genes were potential drug targets. GO analysis revealed 856 biological processes (BP), 139 molecular functions (MF), and 89 cellular components (CC). The KEGG pathway enrichment analysis indicated that the lipid level and atherosclerosis might influence the treatment of dyslipidemia. Molecular docking showed that quercetin bound well to CCND1, HIF1A, MYC, AKT1, and EGFR genes. These findings were in accord with the prediction obtained through the network pharmacology approach.

**Conclusions:**

This study revealed the primary pharmacological effects and relevant mechanisms of the SZHP in treating dyslipidemia. Our findings may facilitate the development of the SZHP or its active compounds as an alternative therapy for dyslipidemia. Still, more pharmacological experiments are needed for verification.

## 1. Introduction

Dyslipidemia is one of the significant risk factors for cardiovascular diseases (CVD) and seriously affects the quality of life of patients. It usually manifests as raised plasma concentrations of total cholesterol (TC), triglycerides (TG), low-density lipoprotein cholesterol (LDL-C), a decreased plasma concentration of high-density lipoprotein cholesterol (HDL-C), or a combination of the above features [1]. According to the 2019 WHO Global Burden of Disease Study, dyslipidemia that is characterized by elevated levels of LDL-C is the third-largest modifiable risk factor for the global CVD burden, following high systolic blood pressure and high fasting plasma glucose [2]. In 2019, 8.54 million people died of ischemic heart disease (IHD) worldwide, and the death of 3.78 million of them was related to increased plasma levels of LDL-C; 2.73 million people died of ischemic stroke, and the death of 0.61 million of them was related to high plasma levels of LDL-C [3].

The treatment of dyslipidemia is dominated by statins, used as monotherapy or in combination with ezetimibe, proprotein convertase subtilisin/kexin type 9 (PCSK9) inhibitors, and fibrates, which have been demonstrated to be effective in lipid-lowering and cardiovascular risk reduction. Statins act by decreasing the cellular cholesterol content by selectively inhibiting the enzyme HMG-CoA reductase, limiting cholesterol biosynthesis, and lowering hepatic cholesterol concentrations [4]; thus, it reduces major vascular events, major coronary events and coronary revascularizations, and ischemic stroke [5]. However, statins are often associated with adverse effects such as liver toxicity and myopathy. Recent evidence also showed that they are associated with an increased incidence of diabetes [6], which might be related to their effect of decreasing insulin sensitivity by lowering plasma adiponectin levels in a dose-dependent manner, thereby leading to an approximately 20–30% higher risk of diabetes [7–9]. In addition, intensive statin therapy is often associated with a higher incidence of myolysis as well as a higher risk of liver enzyme elevation in the Chinese population [10]. Therefore, alternative therapies are necessitated to reduce adverse effects while maintaining the therapeutic effects.

Traditional Chinese medicine (TCM), often used as a complementary and an alternative therapy, has demonstrated beneficial effects among patients with CVD and has been increasingly welcomed worldwide [11]. It has been used for over a thousand years to treat dyslipidemia in China, with the advantages of a multitarget mechanism, a remarkable effect, and a good safety profile. With various active ingredients, TCM features global regulation of glucose and lipid metabolism as well as energy homeostasis [12].

Crataegi Folium (known as Shanzhaye [SZ], the leaves of *Crataegus pinnatifida* Bge. var. major N.E. Br. or *Crataegus pinnatifida* Bge) and Alisma rhizoma (known as Zexie [ZX], the dried tuber of *Alisma orientale* (Sam.) Juzep or *Alisma plantago-aquatica* Linn.) form a classic combination of herbal drugs and have been widely used in clinical practice for their significant effects in dyslipidemia. According to the theory of TCM and the Pharmacopoeia of the People's Republic of China, SZ activates blood and removes stasis, regulates qi and promotes blood circulation, removes turbidity, and lowers lipids, whereas ZX promotes urination and drains dampness, discharges heat, resolves turbidity, and lowers lipid [13]. Through data mining on clinical studies on the treatment of dyslipidemia with TCM compound prescriptions from 1979 to 2019, Geng et al. found that SZ was the most frequently used herb (frequency: 577, prescription ratio: 73.41%), followed by ZX (frequency: 451, prescription ratio: 57.38%), and SZ and ZX were the most frequently used drug pair (frequency: 378, prescription ratio: 48.09%) [14].

Studies have shown that SZ has a variety of effects on dyslipidemia, diabetes, and atherosclerosis [15], while ZX has good therapeutic effects of lowering lipids, eliminating edema, improving metabolism, as well as reducing inflammation and tumor burden [16]. To date, the hypolipidemic activity and the clinical implications of SZ and ZX extracts have been extensively studied. It was found that flavonoid and triterpenic acids extracted from SZ can decrease the level of serum lipids, such as TC and TG [17, 18]. Furthermore, triterpenes in ZX can effectively lower serum lipids by lowering serum TC and LDL-C levels, thus reducing the Atherogenic Index [19]. Therefore, they have been regarded as the main ingredients in some Chinese patent lipid-lowering drugs such as Xuezhiling Tablets, Zhibitai Capsules, and Zhikeqing Capsules. However, the molecular mechanism of the SZ-ZX herb pair (SZHP) against dyslipidemia still remains poorly understood.

Recently, network pharmacology has been proposed as a promising approach to study TCM from the systems perspective and the molecular level, which integrates a large number of network database resources and uses bioinformatics technology to build an overall drugs-targets-diseases network. In addition, the multicomponent, multitarget, and regulatory network based on network pharmacology can reveal the complex mechanism of the action of the drugs; therefore, this approach is particularly suitable for studying the mechanism of TCM compounds and their complex components from a holistic perspective [20]. Molecular docking simulates in vivo ligand-receptor docking through informatics. As it predicts the potential binding mode between ligands and acceptors, it has been used for in-depth exploration of the relationship between ligands and receptors as well as the design and development of new drugs [21].

**Figure 1:**
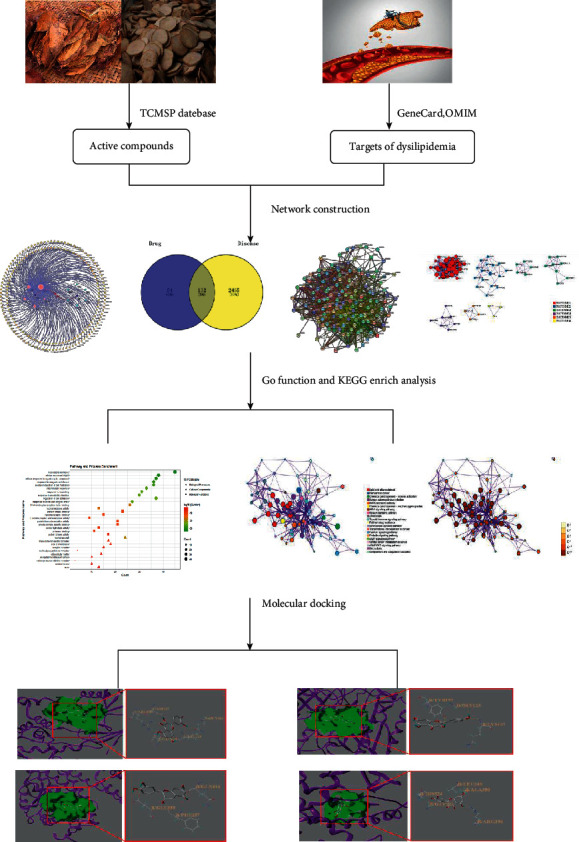
Workflow for network pharmacology-based prediction and molecular docking technology.

Therefore, with the help of network pharmacology and molecular docking technology, this study intends to explore the cotreatment of dyslipidemia with the SZHP and further clarify its molecular mechanism in order to provide a basis for the interpretation of relevant connotations, theoretical research, and precise clinical application of this promising herb pair. The specific network pharmacology and molecular docking technology employed in the study are shown in Figure 1.

## 2. Materials and Methods

### 2.1. Acquisition of Bioactive Compounds of SZHP

The Traditional Chinese Medicine Systems Pharmacology Database and Analysis Platform (TCMSP, https://tcmsp-e.com/, updated version, 2 June 2021) is specifically designed for the identification of the drug-target and drug-disease networks as well as the mechanism of action of TCM [22], and all the candidate herbal compounds of the SZHP needed in this study were obtained from it. Based on literature reports and pharmacokinetic parameters, the pharmacokinetic properties, including absorption, distribution, metabolism, and excretion (ADME), are essential contributors to the bioactivities of drugs. The compounds with an oral bioavailability (OB) of ≥30% are regarded with excellent absorption and slow metabolism after oral administration, and the compounds with a druglikeness (DL) of ≥0.18 were considered chemically suitable for drug development. Hence, two ADME-related parameters, OB and DL, were selected to identify the potentially active compounds in the SZHP [23].

### 2.2. Prediction of Compound-Related Targets

After acquiring the active compounds of the SZHP from the TCMSP, the corresponding targets were obtained. The UniProt database (https://www.uniprot.org/) was used to convert all target proteins into their corresponding gene symbols with the “*Homo sapiens*” species to standardize gene names and organisms to prevent over annotation of similar proteins.

### 2.3. Acquisition of Herb-Disease Genes

We searched GeneCards (https://www.genecards.org/) and the OMIM databases (Online Mendelian Inheritance in Man, https://omim.org/) for “*Homo sapiens*” genes only, with keywords of “dyslipidemia,” “hyperlipidemia,” “hypercholesterolemia,” “hypertriglyceridemia,” “hyperlipoproteinemia,” “hypoalphalipoproteinemia,” and “hypobetalipoproteinemia”; duplicate genes were deleted. All the target genes obtained from the two databases were summarized. Subsequently, active compound targets were mapped to the dyslipidemia-related targets, and therapeutic targets of the SZHP against dyslipidemia were obtained using a Venny 2.1 online tool.

### 2.4. Protein-Protein Interaction (PPI) Network

The common targets of drugs and diseases were entered into the Search Tool of the Retrieval of Interacting Genes database (STRING, https://www.string-db.org/) for PPI network construction. To screen out key target genes with a high degree of connectivity with the SZHP against dyslipidemia, the CytoNCA tool (a plug-in of Cytoscape) was used to analyze the topological properties of the targets. Topological analysis was performed using a network analyzer tool for the estimation of three parameters, i.e., degree centrality (DC), betweenness centrality (BC), and closeness centrality (CC), with a greater node connection degree centrality indicating higher importance of a node in the PPI network. Targets showing above-average values of the three parameters were selected by the value of DC.

### 2.5. Protein-Protein Interaction Enrichment Analysis

Protein-protein interaction enrichment analysis has been carried out with the use of the following databases: STRING, BioGrid, OmniPath, and InWebIM. Only physical interactions in STRING (physical score >0.132) and BioGrid databases were used. The resultant network contained the subset of proteins that formed physical interactions with at least one other member in the list. If the network contained 3 to 500 proteins, the molecular complex detection (MCODE) algorithm was to be applied to identify densely connected network components.

### 2.6. Gene Ontology (GO) Functional Annotation and Kyoto Encyclopedia of Genes and Genomes (KEGG) Pathway Enrichment Analysis

To further explore the underlying mechanisms of the SZHP for the treatment of dyslipidemia and to elucidate the biological processes of the target proteins in different clusters and their roles in signaling transduction, GO function annotation (https://geneontology.org/) and KEGG pathway enrichment analysis (https://www.genome.jp/kegg/) were used. By entering a list of target genes and limiting the species to human only, all target genes were corrected to their official gene symbol. GO terms and KEGG pathways with a *p* value of <0.01 after Bonferroni correction were considered significant; to avoid false positiveness, Benjamini–Hochberg's FDR multiple comparison correction was used, with a minimum count of 3 and an enrichment factor of >1.5 in the KEGG pathway enrichment analysis. Finally, the first 20 entries were selected and plotted into a bar chart for visualization using an online gene function analysis (Metascape, https://metascape.org/).

### 2.7. Compound-Target Molecular Docking Verification

Molecular docking is a crucial technology of network pharmacology that combines known proteins with small compounds and validates compound-target interaction [24]. Using the standard model of the Surflex-Dock module of the SYBYL 2.1.1 software, the three core bioactive compounds of the SZHP were molecularly docked with key targets. First, the format file of the main active ingredient mol2 was downloaded from the PubChem database in advance, and then minimized using the ChemDraw20.0 software. Secondly, the ID and highest resolution 3D structure of the target protein were found in the PDB database (https://www.rcsb.org/) and exported in the PDB format. Finally, the water and small molecule ligands were removed using the SYBYL software, and the terminal residue repair, hydrogenation, and charge calculation were performed to complete the molecular docking.

## 3. Results

### 3.1. Screening of Active Compounds of the SZHP

**Figure 2:**
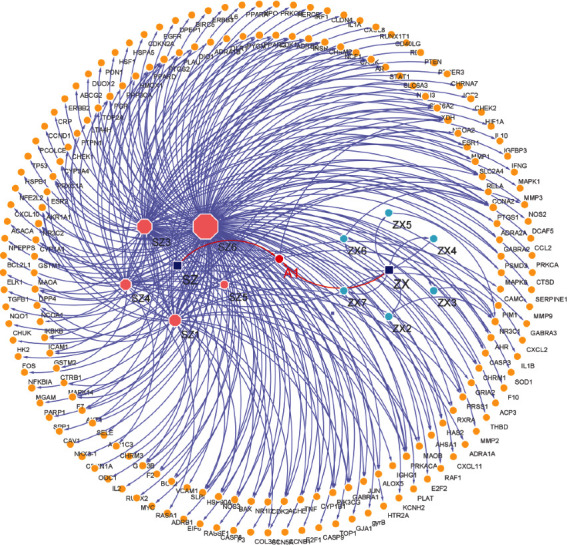
Compound-target network of the SZHP. The yellow circle represents genes, the aqua and light salmon colors represent the active compounds of the SZHP, and the red circle represents the common compounds of the SZHP.

After ADME screening on the TCMSP database, 12 candidate active compounds and 186 targets were screened out (Table 1). The results indicated that one compound regulated multiple targets, such as quercetin, kaempferol, sitosterol, and isorhamnetin, suggesting that the SZHP has a multi-ingredient and multitarget feature in the treatment of dyslipidemia. Besides, a compound might be present in different herbs, while different herbs could contain multiple effective components, which is an essential basis for the multitarget effect of TCM. Then, the corresponding genes were extracted in the Uniprot database through checking of targets. Active compounds and target genes are presented in Figure 2.

### 3.2. Drug-Disease Core Target Acquisition

**Figure 3:**
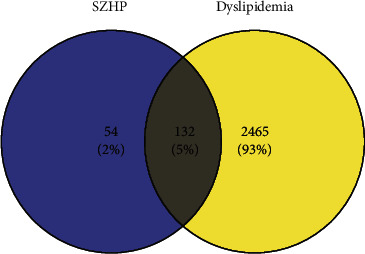
The Venn diagram shows 2,597 targets screened out from databases, which included 186 targets of the SZHP. The intersection indicates the 132 targets of the SZHP for dyslipidemia.

A total of 2,255 related genes were retrieved from the GeneCards database, and 493 genes were obtained from the OMIM database. After merging and deduplicating the genes, 2,597 genes were obtained. A Venn diagram was constructed by inputting the screened drug targets and disease targets into the Venny software, and a total of 132 shared targets were obtained. Details are presented in Figure 3.

### 3.3. PPI Network Construction and Topology Analysis of Drug-Disease-Targets

**Figure 4:**
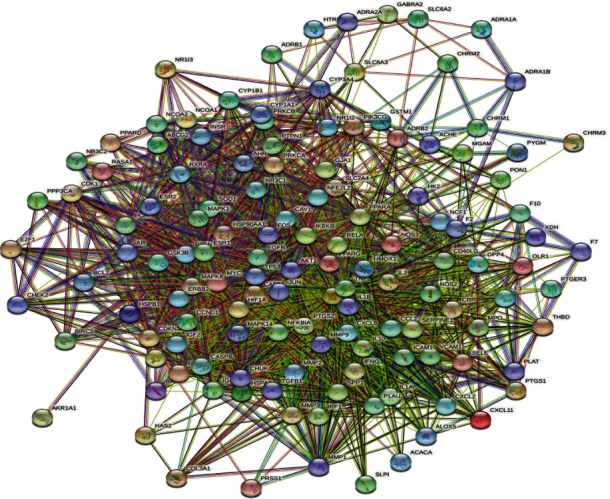
PPI network of common targets of the SZHP and dyslipidemia.

The common targets of drugs and diseases were entered into the STRING database to construct a PPI network (Figure 4), which was then imported into Cytoscape for topology analysis using the network analyzer tool. The network comprised 132 nodes and 2,368 edges, with an average connection degree of 35.88. The three parameters of DC, BC, and CC were used as reference standards, and the genes with scores greater than the average were selected as key targets through the ranking of connectivity values; finally, a total of 23 key targets were screened. The 10 targets with the highest values are presented in Table 2.

### 3.4. GO Enrichment Analysis

**Figure 5:**
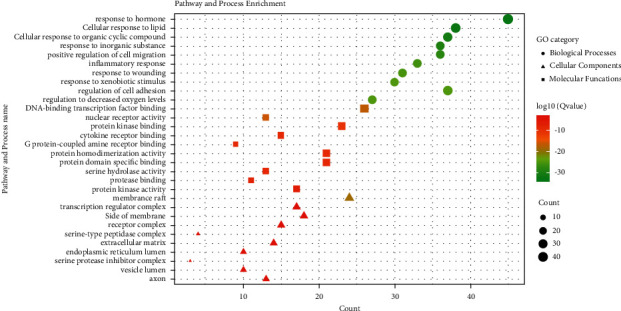
BP, CC, and MF of GO enrichment analysis.

A total of 856 biological processes (BP), 139 molecular functions (MF), and 89 cellular components (CC) were enriched. Most GO terms of BP were related to response to hormones, cellular response to lipids, cellular response to organic cyclic compounds, response to inorganic substances, positive regulation of cell migration, inflammatory response, response to wounds, response to xenobiotic stimuli, regulation of cell adhesion, response to decreased oxygen levels, etc. The main terms of CC were associated with the binding to DNA-binding transcription factors, nuclear receptor activity, protein kinase binding, cytokine receptor binding, G protein-coupled amine receptor activity, protein homodimerization activity, protein domain-specific binding, serine hydrolase activity, protease binding, protein kinase activity, etc. MF enrichment was mainly involved in membrane raft, transcription regulator complex, side of the membrane, receptor complex, serine-type peptidase complex, extracellular matrix, endoplasmic reticulum matrix, serine protease inhibitor complex, vesicle lumen, axons, etc. The top 10 terms of BP, CC, and MF were ranked based on their *Q*-value, which are presented in Figure 5, with higher *Q*-values and green color indicating greater enrichment of the GO terms.

### 3.5. KEGG Pathway Enrichment

**Figure 6:**
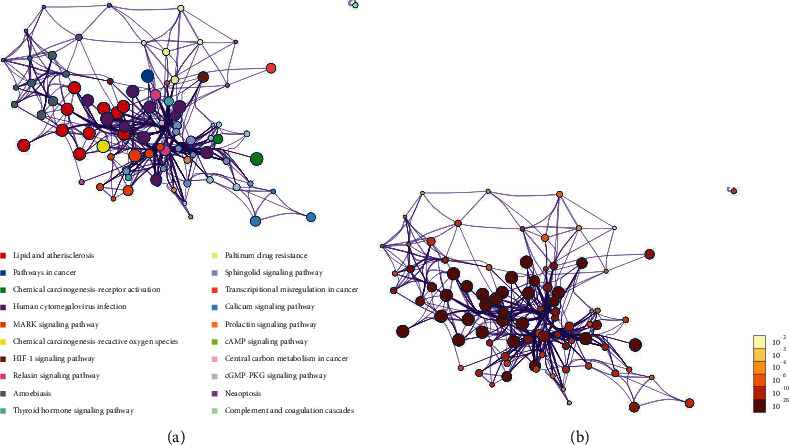
The top 20 enriched KEGG pathways: (a) colored by cluster ID, where nodes sharing the same cluster ID are typically close to each other; (b) colored by *p* value, where terms containing more genes tended to have a more significant *p* value.

The KEGG pathway enrichment showed that a total of 35 signaling pathways were enriched after FDR correction, with the first 20 pathways including lipid and atherosclerosis, pathways in cancers, chemical carcinogenesis-receptor activation, human cytomegalovirus infection, MAPK signaling pathway, chemical carcinogenesis-reactive oxygen species, HIF-1 signaling pathway, relaxin signaling pathway, amoebiasis, and thyroid hormone signaling pathway. After hierarchical clustering of the enrichment results, the network with a similarity of >0.3 was considered a sub-network. The most significant pathway was used as the representative of the enriched networks (Figure 6).

### 3.6. Protein-Protein Interaction Pathway and Process in Enrichment MCODE Analysis

**Figure 7:**
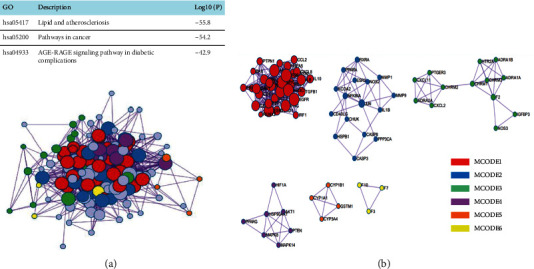
(a) The top 3 highest-scoring terms in the enrichment MCODE analysis; (b) the top 6 gene clusters in the enrichment MCODE analysis.

Key genes were screened out through MCODE analysis, network construction, and MCODE analysis in the total dataset of STRING, OmniPath, InWebIM, and BioGrid for the independent enrichment analysis of gene clusters. The pathway and process enrichment analysis has been independently applied to each MCODE component. The three highest-scoring terms by the *p* value have been retained as the functional description of the corresponding components, as shown in Figure 7 and Table 3.

### 3.7. Molecular Docking Verification

**Figure 8:**
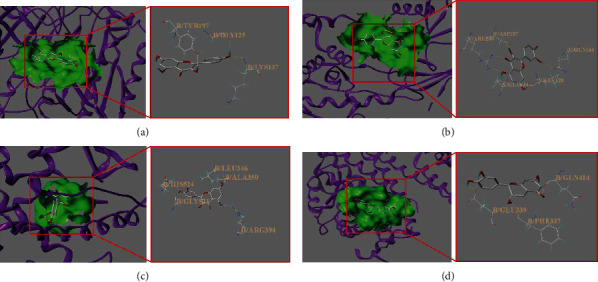
Molecular models of active compounds binding to the predicted targets. (a) CASP3 and kaempferol; (b) HIF-1 and quercetin; (c) ESR1 and isorhamnetin; (d) ESR1 and ent-Epicatechin.

The SYBYL 2.1.1 software was used to verify the molecular docking of some important active ingredients. Based on the protein binding pocket of the target proteins and ligands, the docking effect was evaluated using the function of the total score (TS), which simulated the binding capacity in the unit of the negative logarithm and converted the binding-free energy formula (∆*G* = −2.303 RT × TS, where *R* is the ideal gas constant of the molecule and *T* is the thermodynamic temperature of the ideal gas), with a greater total score indicating higher stability of the ligand-receptor binding. It is generally recognized that a total score greater than 4.25 indicates fair binding activity between the active component and the target, a score greater than 5.0 indicates good binding activity, and a score greater than 7.0 indicates excellent binding activity [25]. The results showed that approximately 92% of them exhibited binding abilities, and 69% exhibited good binding abilities, which indirectly verified that the SZHP had a regulatory effect on dyslipidemia. At the same time, the above molecular docking results were consistent with those of the previous network screening, which demonstrated the reliability of network pharmacology. Some detailed compound-target interactions are shown in Table 4, and the docking simulation is shown in Figure 8.

## 4. Discussion

Dyslipidemia is an independent risk factor of cardiovascular diseases. Its harm is insidious, gradual but systemic, and may cause damage to the heart, brain, kidney, and other vital organs, leading to diseases such as atherosclerosis, coronary heart disease, and stroke. With the increasing prevalence and morbidity, dyslipidemia has become a critical global concern for public health. SZ and ZX are well-known traditional medicinal herbs and have shown a variety of biological and pharmacological activities, such as anti-inflammatory, antioxidant, and hypolipidemic effects. To investigate the underlying mechanism of the action of the SZHP in the treatment of dyslipidemia, this study was conducted using the network pharmacology approach.

In this study, we found that the core active components of the SZHP in the treatment of dyslipidemia included sitosterol, stigmasterol, isorhamnetin, kaempferol, quercetin, Alisol B, Alisol B monoacetate, etc. Stigmasterol and sitosterol belong to phytosterols, which have similar chemical structures with cholesterol and are known to decrease the circulating cholesterol levels by competing with cholesterol for intestinal absorption or regulating proteins involved in cholesterol metabolism [26]; therefore, these components significantly lower blood levels of TC, TG, and LDL-C and increase the ratio of HDL-C/TC and HDL-C/LDL-C, thereby reducing the Arteriosclerosis Index and relieving dyslipidemia [27, 28]. Used at a dose suggested for humans by the FDA, stigmasterol can reduce the reabsorption or absorption of cholesterol, bile acids, and dietary lipids, thus resulting in the attenuation of weight gain and reduction of hepatic TG accumulation [29]. Furthermore, stigmasterol can also inhibit the activity of SREBP2, thereby reducing the production of cholesterol [30], while sitosterol can reduce the expression of NPC1L1 and inhibit the absorption of cholesterol [31]. Quercetin and kaempferol are flavonoid monomers of flavonols; several studies have shown that quercetin can reduce plasma levels of TC, TG, LDL-C, very low-density lipoprotein cholesterol (VLDL-C), and free fatty acids (FFAs), as well as increase HDL-C and adiponectin levels [32–36]. They also inhibit the formation of foam cells by activating the PPAR*γ*-ABCA1 pathway and thereby improving lipid levels [37, 38]. Kaempferol can inhibit Akt activity, reduce TG levels, as well as induce hepatocyte autophagy and reduce lipid droplet formation in mouse liver by attenuating the Akt/mTOR pathway and activating PPARs [39]. Zhang et al. found that isorhamnetin was a dietary source of the PPAR*γ* antagonist, which reduced body weight, ameliorated insulin resistance, and alleviated hepatic steatosis through the suppression of PPAR*γ* transactivity, which was beneficial to obesity and hepatic steatosis [40]. Triterpenoids such as Alisol B, Alisol B monoacetate, and Alisol C monoacetate are abundant in ZX; they have been selected as the main quality markers of ZX, and have also shown good properties [41]. Lipoprotein lipase (LPL) is a typical key lipid metabolism enzyme, and the rate-limiting enzyme of the TG degradation reaction plays a key regulatory role in TG metabolism. It has been reported that, in the metabolism of fat, Alisol B can decrease the level of TG by enhancing the activity of LPL in lysosomes; therefore, it plays a key role in regulating blood lipids [42]. A study found that Alisol B 23-acetate induced decreases in serum and hepatic lipids, which was related to decreased hepatic lipogenesis, through decreasing hepatic the levels of SREBP-1c, fatty acid synthase (FAS), anti-acetyl CoA carboxylase 1 (ACC1), and stearoyl-CoA desaturase 1 (SCD1); it was also associated with increased lipid metabolism as it induced peroxisome proliferator-activated receptor*α* (PPAR*α*), carnitine palmitoyl transferase 1*α* (CPT1*α*) acyl-coenzyme A dehydrogenase (ACADS), and LPL [43]. 1-Monolinolein not only inhibits the expression of apolipoprotein CIII (apo CIII) by inhibiting the transport and absorption of cholesterol, but also inhibits the expression of lipoprotein-related phospholipase A2 (Lp-PLA2), thereby reducing the generation of free fatty acids [44]. Taken together, the main active components of the SZHP could regulate multiple signaling pathways, thereby regulating lipid synthesis and metabolism, lowering cholesterol levels, and preventing or delaying the development of dyslipidemia.

The common target PPI network analysis showed that the key targets of the SZHP in the treatment of dyslipidemia included CCND1, CASP3, HIF1A, ESR1, ERBB2, MYC, PTEN, TP53, AKT1, and EGFR. Caspase-3 is a protein in the caspase family that can cause an apoptotic cascade. Studies showed that hyperlipidemia could enhance the peroxidation of lipids and the formation of modified oxidative low-density lipoprotein (ox-LDL), which is mediated by caspase-3; it could also increase NF-kB levels, damage endothelial cells, and promote monocyte adhesion; therefore, it is an important link in the development from hyperlipidemia to AS [45–47]. HIF-1 may regulate the sterol regulatory element binding the protein-1 pathway, which augments lipid biosynthesis. A study showed that partial HIF-1A deletion attenuated hypoxia-induced contractions in fasting serum insulin and liver TG levels, thereby mediating the intermittent hypoxia-induced hyperlipidemia response [48]. The upregulation of HIF-1A plays a protective role in the corpus cavernosum in hypercholesterolemic rats [49]. Estrogen is an important regulator of metabolic homeostasis and lipid metabolism; acting through cognate nuclear hormone receptors, it has specific cardioprotective effects. Estrogen receptor *α* (ESR1) is one of its specific receptors involved in the mediation of the physiological function of estrogen. Polymorphic alterations of ESR1 are associated with conditions such as arterial hypertension, altered serum lipid levels, coronary atherosclerosis, and altered HDL-C levels in menopausal women [50, 51]. The tumor protein p53 (TP53) gene plays a crucial role in lipid metabolism and is involved in normal and pathological conditions. TP53 usually acts as a negative regulator of lipid synthesis by activating fatty acid oxidation and inhibiting FAS [52]. In addition, in mice prone to atherosclerosis, the association between TP53 and atherosclerosis becomes apparent, with lower levels of TP53 associated with larger atherosclerotic plaques [53]. Collectively, the results indicated the multitarget feature of the SZHP in the treatment of hypolipidemic.

GO function analysis showed that the therapeutic effect of the SZHP in dyslipidemia is mainly exhibited as a response to hormone, cellular response to lipid, the binding to DNA-binding transcription factors, etc. The KEGG pathway analysis showed that it was mainly concentrated in lipid and atherosclerosis, MAPK signaling pathway, HIF-1 signaling pathway, and relaxin signaling pathway, while the MCODE analysis showed that pathways in cancer, human cytomegalovirus infection, and the AGE-RAGE signaling pathway in diabetic complications comprised the most correlated gene cluster. The most significant pathways also included the lipid and atherosclerosis pathway and the IL-17 signaling pathway.

It has been well established that lipid metabolism is associated with atherosclerosis. In atherosclerosis, endothelial dysfunction or apoptosis occurs after chronic injury. The normal physiological function and integrity of the intima are affected, resulting in increased infiltration of lipids into the subintima, which is the initial link to atherosclerosis. Lipids, especially LDL, are oxidized and modified to form ox-LDL after entering the subintima, which leads to endothelial damage, local inflammation in the wall of blood vessels, as well as the formation of foam cells and lipid accumulation, thereby resulting in the aggravation of atherosclerosis [54]. The MAPK signaling pathway regulates the inflammatory response in atherosclerosis, which is a cascade constituted by a series of protein kinases and their phosphorylation effects. Subsequently, it leads to the activation of the transcription factor NF-kB, thereby regulating the expression of inflammatory cytokines [55]. Studies have confirmed that a high-fat diet can activate the HIF-1 signaling pathway, promote the expression of hypoxia-inducible factor-1 (HIF-1) in the hypothalamus to increase the basal metabolic rate, and act as a protective factor to reduce the impact of hypothalamic dysfunction on the hypothalamic and systemic regulation of metabolism and energy homeostasis [56]. Similar to the HIF-1 signaling pathway, a high-fat and high-sugar diet also increases advanced glycation end products (AGEs) [57]. Hyperlipidemia may activate the AGE-RAGE signaling pathways including NADPH, MAPK, and ERK signal transduction cascades caused by p38 and protein kinase C, induce cell damage and inflammatory responses, and ultimately lead to the formation of atherosclerosis [58, 59]. Suchal et al. [60] demonstrated that kaempferol attenuated the myocardial ischemia-reperfusion injury in diabetic rats by reducing AGE-RAGE/MAPK-induced oxidative stress and inflammation. IL-17 is a proinflammatory cytokine that promotes endothelial cell activation through the p38MAPK pathway [61], increases the expression of the IL-17A receptor in apoE knockout mice [62], and reduces atherosclerotic lesions. Acting along with hypoxia, IL-17 also promotes the migration and invasion of proinflammatory cells [63]. Studies showed that, through IL-17, PCSK9 could mediate the cross-linking of hyperlipidemia, atherosclerosis, and immune responses [64].

Hence, molecular docking was performed to verify further molecular mechanisms of the SZHP treating dyslipidemia. The results indicated that quercetin had an excellent binding activity with many targets, including CCND1, HIF1A, MYC, AKT1, and EGFR; therefore, it might be a key compound for the effect against dyslipidemia. The above findings were also in line with the results of the network pharmacology analysis. These results might be a significant guidance for further research to determine the molecular targets of the SZHP in the treatment of dyslipidemia as well as for applications of network pharmacology in drug development. It may also provide insights for the subsequent development of novel TCM lipid-lowering drugs with high efficiency, low toxicity, and multiple targets, in order to reduce the mortality of cardiovascular diseases.

### 4.1. Limitations

Despite the strength shown in the present study, some limitations need to be noted. First, the public databases investigated in the study are constantly updated; thus, some other bioactive ingredients and target genes might not have been included in our analysis. Second, the current research on the extraction of the main components of TCM and its drug-like properties is still insufficient, and the important targets have been identified mainly based on computer simulation and verification against existing studies on drugs. Therefore, further studies on the pharmacology of traditional Chinese medicine are still needed.

## 5. Conclusion

In the present study, a network pharmacology approach in combination with molecular docking was used to explore the targets, mechanism, related signal pathways of the SZHP against dyslipidemia, as well as the binding abilities of herbal ingredients and their targets. The results showed that quercetin, kaempferol, isorhamnetin, Alisol B, etc. were potential active ingredients of the SZHP, and they act on key targets such as CCND1, CASP3, HIF1A, and ESR1 to regulate a variety of biological processes, molecular functions, cellular components, and pathways. This study not only revealed that the SZHP regulates lipid metabolism comprehensively through multiple components, multiple targets, and multiple pathways, but also provided a theoretical basis for the ameliorative effect of the SZHP against dyslipidemia. Hopefully, this study may provide new ideas for the development of the SZHP or its active components as an alternative therapy for dyslipidemia.

## Figures and Tables

**Table 1:** Active compounds of SZHP.

MOL ID	Active compound	OB (%)	DL	Drug	Serial no.
MOL000359	Sitosterol	36.91390583	0.7512	SZ, ZX	A1
MOL000354	Isorhamnetin	49.60437705	0.306	SZ	SZ1
MOL000422	Kaempferol	41.88224954	0.24066	SZ	SZ3
MOL000449	Stigmasterol	43.82985158	0.75665	SZ	SZ4
MOL000073	Ent-Epicatechin	48.95984114	0.24162	SZ	SZ5
MOL000098	Quercetin	46.43334812	0.27525	SZ	SZ6
MOL000831	Alisol B monoacetate	35.57623621	0.80629	ZX	ZX2
MOL000849	16*β*-Methoxyalisol B monoacetate	32.42724106	0.7679	ZX	ZX3
MOL000853	Alisol B	36.76038067	0.81993	ZX	ZX4
MOL000856	Alisol C monoacetate	33.06358947	0.82763	ZX	ZX5
MOL002464	1-Monolinolein	37.17662836	0.30249	ZX	ZX6
MOL000862	[(1S, 3R)-1-[(2R)-3, 3-Dimethyloxiran-2-yl]-3-[(5R, 8S, 9S, 10S, 11S, 14R)-11-hydroxy-4, 4, 8, 10, 14-pentamethyl-3-oxo-1, 2, 5, 6, 7, 9, 11, 12, 15, 16-decahydrocyclopenta [a] phenanthren-17-yl] butyl] acetate	35.58	0.81	ZX	ZX7

**Table 2:** Top 10 key targets of dyslipidemia.

Targets	DC	BC	CC
CCND1	98	0.007020409	0.648514851
CASP3	76	0.017834894	0.708108108
HIF1A	75	0.011567789	0.685863874
ESR1	73	0.019366461	0.678756477
ERBB2	71	0.007947206	0.623809524
MYC	69	0.010977465	0.668367347
PTEN	69	0.013298088	0.655
TP53	66	0.023982438	0.735955056
AKT1	66	0.071732181	0.803680982
EGFR	65	0.022700048	0.693121693

**Table 3:** Pathway and process of protein-protein interaction in the enrichment MCODE analysis.

MCODE	GO	Description	Log 10 (*p*)
MCODE1	Hsa05200	Pathways in cancer	−30.6
	Hsa05163	Human cytomegalovirus infection	−25.3
	Hsa04933	AGE-RAGE signaling pathway in diabetic complications	−23.9
MCODE2	Hsa05417	Lipid and atherosclerosis	−20.1
	Hsa04657	IL-17 signaling pathway	−16.1
	Hsa05200	Pathways in cancer	−13.7
MCODE3	Hsa04080	Neuroactive ligand-receptor interaction pathway	−14.6
	Go: 0007200	Phospholipase C-activating G protein-coupled receptor signaling pathway	−14.3
	Go: 0007188	Adenylate cyclase-modulating G protein-coupled receptor signaling pathway	−13.9
MCODE4	Hsa05200	Pathways in cancer	−9.7
	Hsa05417	Lipid and atherosclerosis	−9.4
	Hsa05208	Chemical carcinogenesis—reactive oxygen species	−9.4
MCODE5	Hsa05204	Chemical carcinogenesis—DNA adducts	−10.6
	Hsa00980	Metabolism of xenobiotics by cytochrome P450	−10.4
	Go: 0006805	Xenobiotic metabolic process	−9.8
MCODE6	Hsa04610	Complement and coagulation cascades	−7.7
	Go: 0051897	Positive regulation of protein kinase B signalling	−7.2
	Go: 0007596	Blood coagulation	−6.7

**Table 4:** The binding ability between active components and core targets.

Target	Active compound	Structure identifier	Total score	Crash	Polar	Cs score
CCND1	Quercetin	2W96	5.1903	−1.0717	4.1885	4
CASP3	Quercetin	1NMS	4.9902	−0.882	2.8775	4
	Kaempferol	1NMS	5.2574	−1.0301	3.0597	5
HIF1A	Quercetin	6GMR	6.6632	−1.7108	6.7477	5
ESR1	Isorhamnetin	1L2I	6.7808	−2.1481	3.3145	5
	Ent-epicatechin	1L2I	5.5575	−1.8304	4.3591	5
ERBB2	Quercetin	1MFG	4.5064	−0.4128	4.4425	5
MYC	Quercetin	1NKP	6.3422	−0.8011	2.2238	4
PTEN	Quercetin	1D5R	4.2476	−1.0926	3.5241	5
TP53	Quercetin	1AIE	4.3401	−0.9861	2.7229	5
AKT1	Quercetin	1UNQ	5.0166	−0.7164	4.8684	5
	Kaempferol	1UNQ	5.0044	−0.6681	4.872	3
EGFR	Quercetin	1XKK	5.7674	−0.3837	1.8907	4

## Data Availability

The data used to support the findings of this study are available from the corresponding author upon request.
